# Reliability of observer ratings: Qualitative behaviour assessments of shelter dogs using a fixed list of descriptors

**DOI:** 10.1016/j.vas.2020.100145

**Published:** 2020-10-01

**Authors:** Solveig Marie Stubsjøen, Randi Oppermann Moe, Kristin Bruland, Tuva Lien, Karianne Muri

**Affiliations:** a Norwegian Veterinary Institute, Department of Animal Health, Welfare and Food Safety, Section for Terrestial Animal Health, Wildlife and Welfare, P.O. Box 750 Sentrum, N-0106 Oslo, Norway; b Norwegian University of Life Sciences, Faculty of Veterinary Medicine, P.O. Box 369 Sentrum, N-0102 Oslo, Norway; c Hegdehaugsveien 3a, N-0352 Oslo, Norway

**Keywords:** Animal welfare, Behaviour, Inter-observer reliability, Qualitative behaviour assessments, Shelter dogs

## Abstract

Qualitative Behaviour Assessment (QBA) is a whole-animal approach used to quantify the expressive style of animals’ behaviour. The aim of this study was to evaluate the inter-observer reliability of QBA of shelter dogs using a fixed list of descriptors. The fixed list of 20 terms was generated using a group of experts and literature reviews. In the pilot study, seven veterinary students scored 12 two-minute video clips, and in the main study, 22 final year veterinary nurse students and third-year veterinary students scored the same videos. The two datasets were analysed using Principal Component Analysis (PCA), and the level of agreement for the main components and individual terms was assessed using Kendall's coefficient of concordance (*W*). In the pilot study, the observer agreement was 0.89 for PC1 and 0.78 for PC2, indicating high inter-observer agreement. The reliability was similarly high for both components in the main study (0.88 and 0.79, respectively). Results also demonstrated high or moderate agreement for most of the terms included in the fixed list. We propose that this approach can be a useful learning tool for students. Our results support further exploration of this method for the assessment of shelter dog welfare by direct observation.

## 1 Introduction

Animal welfare is a societal concern, and there is a need for science-based approaches to improve and document the welfare of both farm and companion animals. All over the world, a large number of dogs live in shelters where the aim is to rehome them and by doing so, optimising their long-term welfare (Walker, Dale, D'Eath & Wemeldsfelder, 2016). However, the shelter environment can in itself be stressful and have negative impacts on the dogs’ welfare (Righi et al., 2019), and there are growing efforts worldwide to develop scientifically based indicators to assess shelter dog welfare (Arena, Wemelsfelder, Messori, Ferri & Barnard, 2019). Welfare indicators should be sensitive not only to animals’ physical health, but also to their mental experience of the conditions in which they live (Wemelsfelder & Mullan, 2014). The presence of positive experiences may be as important for animal welfare as the absence of suffering (Boissy et al., 2007), and there is therefore a need for indicators for both positive and negative welfare states. Previous studies investigating the welfare of dogs in shelters have mainly utilised physiological and behavioural measures of stress (Hennessy et al., 2001; Righi et al., 2019; Rooney, Gaines & Bradshaw, 2007; Titulaer et al., 2013). For instance, cortisol has been measured in both plasma and saliva in order to assess physiological stress responses in shelter dogs (Hennessy, 2013), but these measurements may be influenced by e.g. circadian rhythms and handling stress. Quantitative measurements of behaviour are time consuming, and the significance of highly important but infrequent behaviours can be lost in statistical analyses (Walker et al., 2016).

Charles Darwin postulated in “The expression of the emotions in man and animals” that “*man and animals express the same state of mind by the same movements*” (Darwin, 1872). Some recent studies have included qualitative measures to assess behaviour and welfare of shelter dogs (Berteselli et al., 2019; Menchetti et al., 2019), which may provide important additional information integrating complex behavioural patterns over time (Meagher, 2009). For instance, Menchetti et al. (2019) developed a welfare assessment system to help shelter staff in decision-making processes. The form used for behavioural assessments contained a qualitative score for the overall stress level and five descriptors of dog behavioural traits. Facial expressions in dogs have been investigated using the Dog Facial Action Coding System, which is an anatomically based facial expression coding system that identifies observable facial changes associated with underlying muscle movement (Kaminski, Hynds, Morris & Waller, 2017). A behaviour-based composite scale was developed to assess acute pain in dogs using a predefined list of expressions (Holton, Reid, Scott, Pawson & Nolan, 2001). Kiddie and Collins (2014) developed a quality of life (QoL) assessment tool for kennelled dogs based on behavioural indicators of both positive and negative emotional states, including measures such as “high level of activity” and “listlessness”. The dogs were first observed from a distance, and the assessment subsequently involved the observer approaching, handling, and initiating play with the dog while its behavioural responses were recorded on binary scales.

Another method that may be useful to assess the expressive qualities of an animal's demeanour is Qualitative Behaviour Assessment (QBA). This is an integrative, whole-animal approach used to quantify animals’ expressive style of behaviours (Wemelsfelder & Lawrence, 2001). The QBA method integrates and summarises the animal's dynamic style of interaction with the environment using a list of qualitative descriptors such as *relaxed, uncomfortable, sociable* and *depressed* (Wemelsfelder & Lawrence, 2001). The descriptors have an expressive, emotional connotation and can either be generated by the individual assessors, as in Free Choice Profiling (FCP), or be predefined by researchers, who provide the assessors with a fixed list of descriptors (Andreasen, Wemelsfelder, Sandøe & Forkman, 2013). Visual analogue scales (VAS) ranging from Minimum to Maximum are used to score the behaviour of individuals or groups of animals. Minimum is defined as the level where an expression is not present in/amongst the observed animal(s) at all, whereas Maximum is the level at which the expression is dominant in/across the animal/entire group being observed. Principal component analysis (PCA) is used to analyse data from QBA using fixed lists. With this statistical approach, the number of variables is reduced to a few (usually two) main components, each comprising correlated, and to some degree overlapping, behavioural expressions. The terms that best describe the anchor points at each end are used for interpretation and description of the main components.

QBA can either be applied retrospectively, by assessing animals on video footage, or can be used in field conditions, by direct observations of the animals. QBA is most useful when integrated with other indicators of health and welfare, to form complete welfare assessment protocols (Wemelsfelder & Mullan, 2014). The method has been incorporated into the welfare assessment protocols for farm and working animals, such as cattle, pigs, poultry, sheep, goats, horses and donkeys (e.g. the Welfare Quality® assessment protocol for cattle (Welfare Quality® 2009) and the AWIN welfare assessment protocol for sheep (AWIN, 2015), Minero et al., 2016). In the comprehensive Welfare Quality® protocols, QBA is the only measure that captures positive aspects of animal welfare such as being positively engaged, active and alert (Keeling, Evans, Forkman & Kjaernes, 2013). The Shelter Quality protocol (Barnard et al., 2015) for the assessment of dog welfare in long-term shelters, is also based on the four welfare principles described by the Welfare Quality® project (i.e. good housing, good feeding, good health and appropriate behaviour), and has included QBA as one of the animal-based indicators.

Essential requirements for all methods employed in the assessment of animal welfare are that they are valid, reliable and feasible. The validity of the QBA method can be investigated by assessing how individual QBA descriptors (Muri, Stubsjøen & Valle, 2013; Phythian, Michaelopoulou, Cripps, Duncan & Wemelsfelder, 2016) or principal components are associated with other welfare indicators. QBA has been found to correlate in a biologically meaningful direction with physiological measures (Rutherford, Donald, Lawrence & Wemelsfelder, 2012; Stockman et al., 2011; Wickham et al., 2012) and health measures (des Roches et al., 2018; Phythian et al., 2016) in farm animals. Inter-observer reliability (IOR) concerns the degree to which measurements performed by multiple raters provide similar results. Previous studies have found varying levels of reliability when QBA has been performed using fixed lists, e.g. for cattle (Bokkers, De Vries, Antonissen & de Boer, 2012), pigs (Czycholl, Beilage, Henning & Krieter, 2017; Duijvesteijn, Benard, Reimert & Camerlink, 2014), sheep (Diaz-Lundahl et al., 2019; Muri & Stubsjøen, 2017; Phythian, Michalopoulou, Duncan & Wemelsfelder, 2013), dairy goats (Grosso et al., 2016), and donkeys (Minero et al., 2016). Some studies found satisfactory agreement between observers when scoring videos, but reached lower inter-observer agreement when QBA was performed on-farm (Czycholl et al., 2017; Muri & Stubsjøen, 2017). As for all observer ratings, thorough training and repeated calibration is essential to obtain satisfactory inter-observer reliability for QBA (Tuyttens et al., 2014). The fixed-list approach is more feasible than FCP, and therefore commonly used for on-farm assessments (e.g. in the Welfare Quality® and AWIN protocols).

Walker et al. (2010) used the FCP approach for QBA of working dogs in a standardised setting, and found a high inter-observer reliability. In a more recent study, Walker et al. (2016) found that both qualitative and quantitative methods were able to extract key differences amongst dogs in different housing environments (short- and long-term shelter confinement, and domestic living situation). Arena, Wemelsfelder, Messori, Ferri and Barnard (2017) applied the FCP approach to shelter dogs and found a good inter-observer reliability when scoring video recordings. A fixed list of QBA descriptors saves time and the number of observation sessions required, compared to the FCP approach, which could make QBA a useful and feasible tool in the daily monitoring of behaviour in kennelled dogs (Walker et al., 2016). Recently, Arena et al. (2019) developed a fixed list of QBA terms for shelter dogs. The list of 20 terms was developed based on literature search and an expert opinion survey. The video recordings of dogs were obtained in seven different Italian shelters, aiming to capture a variety of dog behavioural expressions. The dogs were recorded for 2 min during one of three scenarios: under normal conditions with no external intervention, in the presence of an unknown person, or in the presence of a familiar person. Eleven participants in a course for dog trainers were recruited as observers, and a good inter-observer reliability was found when the observers used this list to rank video clips. The current study provides a second test of reliability of QBA of shelter dogs under reasonably similar conditions, and using a fixed list developed independently of the Arena et al. (2019) study. Eleven of the descriptors are the same across these two studies, while nine descriptors differ.

The aim of this study was 1) to generate a fixed list of QBA descriptors suitable for shelter dog welfare assessments, and 2) to evaluate the inter-observer reliability of QBA when different groups of students (veterinary and veterinary nurse students) applied the method to video recordings of shelter dogs, using the fixed list of descriptors.

## 2 Materials and methods

### 2.1 Video footage

For the training of students and QBA scorings, we used video recordings of dogs obtained from a shelter in southern Hungary. The shelter is managed by an animal protection organisation, and approximately 250 dogs were kept in the shelter at the time. A written informed consent was obtained from the administration of the shelter prior to the study. The material was recorded by one of the authors (TL) during a period of four months in the autumn of 2017. The dogs were video-recorded in their kennels (with sound) using a smartphone (Apple iPhone 5), with minimum disturbance of the animals. The final material included 57 video recordings of dogs representing a large variety of morphology, size, age and sex. From this material, fifteen video clips were selected and cut to approximately two minutes length using the Free Video Dub editing program, and were subsequently used to test the inter-observer reliability. The number of dogs housed in each kennel ranged from one to five. These clips were selected with the aim of covering as many aspects of the expressive repertoire of dogs as possible, so as to test inter-observer reliability on wide-ranging patterns of behaviours and expressions.

### 2.2 List of descriptors

A literature review was performed, and qualitative descriptors were selected from relevant QBA studies using free choice profiling or the fixed list approach to assess welfare in different species, and specifically in dogs (Arena et al., 2017; Walker et al., 2010, 2016). In addition, qualitative descriptors considered relevant to the obtained video recordings were selected. A group of experts consisting of veterinarians and an ethologist, all with previous experience with dog behaviour and/or QBA, were involved in generating the list of descriptors. Some of the selected descriptors were modified based on the groups’ discussions about their meaning and importance. Terms reflecting both positive and negative mental states were included. The participants in the group watched two minute video clips of shelter dogs, and used the preliminary list of 20 descriptors to score them. These scores were used as a basis for discussing the assessments following each video clip. Some further modifications were made to the preliminary list, based on the group's discussion, which resulted in the following list of terms (corresponding Norwegian term in brackets): *content* (tilfreds), *uncomfortable* (ukomfortabel), *playful* (leken), *depressed* (nedstemt), *relaxed* (avslappet), *restless* (urolig), *alert* (oppmerksom), *bored* (kjeder seg), *sociable* (sosial), *nervous* (nervøs), *expectant* (forventningsfull), *hesitant* (avventende), *trustful* (tillitsfull), *aggressive* (aggressiv), *energetic* (energisk), *frustrated* (frustrert), *curious* (nysgjerrig), *calming* (konfliktdempende), *indifferent* (nøytral), *stressed* (stresset). The definitions for individual behavioural terms are described in Table 1.

**Table 1:** The fixed list of individual behavioural terms and their written definitions as used by observers to assess shelter dogs in 12 video clips.

Terms	Definitions
Content	Satisfied, positive activity (eg. play, affiliative behaviours), relaxed
Uncomfortable	Uneasy, depressed, may be in pain
Playful	Actively engaged in play, inviting others to play, happy, may vocalise and jump
Depressed	Unresponsive, not interested in/ unwilling to interact with its environment, resigned, empty stare, apathetic, may be in pain
Relaxed	No vocalisation, interested in its surroundings, not nervous, may move around in a relaxed manner or lie down, not depressed
Restless	Impatient, jittery, move around excessively, may vocalise, may seek attention. Play is not included.
Alert	Attentive, eager, actively interested
Bored	Inactive, uninterested, passive
Sociable	Seeks for contact/ interaction, friendly, positive interaction with other dogs
Nervous	Unsure, shy, fearful, may have the tail tucked under the abdomen, may vocalise
Expectant	Alert, wagging the tail, may vocalise, focused, may be restless
Hesitant	Reluctant, withdrawn, vigilant
Trustful	Familiar, affectionate, friendly, seeks attention
Aggressive	May vocalise, shows signs and posture of defensive or offensive aggression
Energetic	Active, may vocalise, insistent
Frustrated	Conflict behaviour, uneasy, irritable, stressed, may vocalise
Curious	Positively interested, alert, exploring, attentive
Calming	Calming signals (e.g. yawning, licking lips/nose, turn the head away, turning the side of the body towards other dog, tail in a low position, ears back, sniffing the ground)
Indifferent	Does not seek contact/interaction, does not vocalise, uninterested, not depressed
Stressed	Nervous, uneasy, may show repetitive (stereotypic) behaviour

### 2.3 Qualitative Behaviour Assessments

The list was subsequently tested in a pilot study on a group of seven senior veterinary students at the Norwegian University of Life Sciences, Faculty of Veterinary Medicine (three students in their fourth year of study, and four students in their fifth/final year of the study) that had consented to participate. On the test day, the students were informed about the purpose and background of the study, the concept of QBA and how to use visual analogue scales (VAS). After the introduction (~1 hour), the students were shown three video clips. The observers should have a common understanding of the terms in use, and they were therefore encouraged to discuss their interpretation of the animals’ behavioural expressions and to compare their results during the training session, with the aim of reaching consensus about the meaning of each term. Subsequently, 12 new video-clips were shown, and the students used the fixed list to give a score for each of the 20 behavioural descriptors. The written definitions of individual terms were used by the observers during the scoring as a reminder of the agreed meaning of each term. They were instructed not to discuss or compare their results during the scoring sessions.

The main study was conducted with final year veterinary nurse students and third year veterinary students at the Norwegian University of Life Sciences, Faculty of Veterinary Medicine, as a practical in the Animal Welfare, Ethology and Ethics course. In addition to the research objective, the practical was also designed to meet two educational goals: 1) to learn and practice this method for assessing animal behaviour and welfare, and 2) to raise the students’ awareness about the importance of validity, reliability and feasibility of animal welfare indicators. The students were informed two weeks prior to the practical that volunteers were sought to participate in the study, and a paper (Muri & Stubsjøen, 2017) was provided in order to present the QBA method and highlight the importance of testing the reliability of scientific methods. Ten veterinary nurse students and twelve veterinary students consented to participate in the study. They were informed that they could use a nickname on the scoring sheets in order to be anonymous.

On the test day, all the students first received a lecture about animal welfare assessments, including a description of welfare assessment protocols, and the pros and cons of animal- and resource-based welfare indicators. In the following practical, they were informed about the purpose and background of the study. The students were then introduced to the concept of QBA, and to visual analogue scales (VAS) and how to use them. After the theoretical introduction, which lasted about one hour, the students were shown three video clips, each lasting approximately two minutes, and were subsequently encouraged to discuss their interpretation of the animals’ behavioural expressions and to compare their results. The students were then shown 12 two-minute video-clips that they had not seen previously. They used the fixed list of descriptors and the written definitions of individual terms, and were told not to discuss or compare their results. The scoring sheets from the study participants were collected at the end of this session. At the end of the practical, the students were encouraged to discuss the QBA approach and their understanding of the different descriptors in a plenary session.

### 2.4 Statistical analyses

QBA scores for each video were registered by measuring the distance in millimetres between the Minimum point of the visual analogue scale, to the mark made by the observer, thus providing a value between 0 and 125. All data were entered into Microsoft Office Excel® 2010, and statistical analyses were conducted in Stata SE/14.2 (StataCorp, College Station, Texas). The principal component analyses (PCA) was conducted using a correlation matrix (no rotation). A combination of the elbow plot criterion and Kaiser's criterion (Tabachnick, Fidell & Ullman, 2013) was used to determine the number of components to retain. Component scores were calculated for the retained components. The inter-observer reliability of the component scores and the scores of each individual behavioural descriptor was assessed using Kendall's coefficient of concordance (*W*).

## 3 Results

### 3.1 Pilot study

**Fig. 1:**
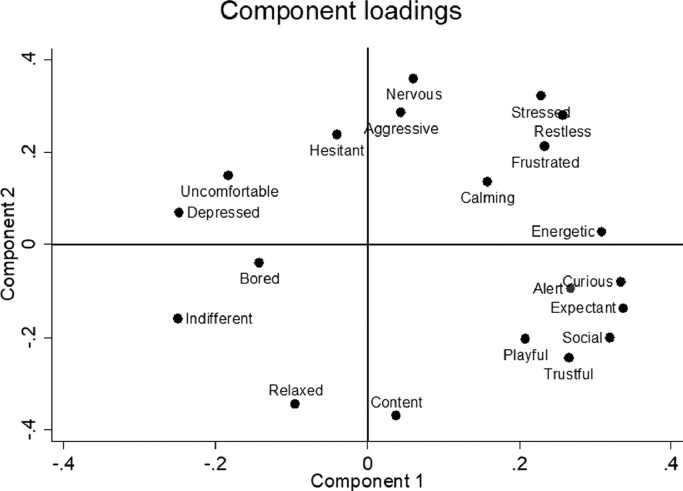
Loading plot depicting how the behavioural terms load along the two main dimensions identified by the principal component analyses of data from the pilot study (12 videos scored by 7 observers).

The principal components analysis of the data from the seven participants in the pilot study resulted in a two-component solution, explaining 33.5% and 22.3% of the variance, respectively. The loading plot in Fig. 1 illustrates the component loadings of each behavioural term across the two components. The first component ranged from *indifferent, depressed, uncomfortable* and *bored* to *curious, energetic, sociable* and *expectant*. The second component ranged from *relaxed, content* and *trustful* to *nervous, stressed, restless* and *aggressive*.

Kendall's coefficient of concordance for the first component (PC1) was 0.89, indicating high inter-observer agreement. The second component (PC2) had a reliability coefficient of 0.78, also indicating high agreement (Table 2).

**Table 2:** Kendall's coefficient of concordance for principal components and individual behavioural terms used by observers in the pilot (*n* = 7 veterinary students) and main study (*n* = 22; 12 veterinary- and 10 veterinary nurse students) to assess shelter dogs in 12 video clips.

Variable	*W* for all students in main study	*W* for vet. students	*W* for vet. nurse students	*W* for pilot study
*PC1*	0.88	0.89	0.88	0.89
*PC2*	0.79	0.81	0.79	0.78
*Content*	0.54	0.53	0.58	0.64
*Uncomfortable*	0.53	0.52	0.59	0.68
*Playful*	0.61	0.66	0.59	0.50
*Depressed*	0.70	0.72	0.70	0.65
*Relaxed*	0.60	0.62	0.62	0.70
*Restless*	0.69	0.74	0.67	0.71
*Alert*	0.72	0.70	0.76	0.58
*Bored*	0.43	0.39	0.53	0.28
*Sociable*	0.71	0.75	0.67	0.83
*Nervous*	0.56	0.57	0.57	0.49
*Expectant*	0.72	0.77	0.70	0.78
*Hesitant*	0.46	0.43	0.54	0.33
*Trustful*	0.59	0.61	0.59	0.71
*Aggressive*	0.29	0.33	0.29	0.36
*Energetic*	0.66	0.66	0.68	0.75
*Frustrated*	0.50	0.58	0.45	0.67
*Curious*	0.64	0.72	0.56	0.75
*Calming*	0.33	0.31	0.41	0.40
*Indifferent*	0.57	0.65	0.53	0.57
*Stressed*	0.58	0.63	0.57	0.75

The seven assessors in the pilot study achieved high agreement (*W* 0.70–0.89) for eight of the behavioural terms (*relaxed, sociable, expectant, trustful, energetic, curious, stressed, restless*), while there was moderate agreement (W: 0.40–0.69) for nine terms (*content, uncomfortable, playful, depressed, alert, nervous, frustrated, calming, indifferent*), and low agreement for the remaining three terms (*bored, hesitant, aggressive*) (Table 2).

### 3.2 Main study

**Fig. 2:**
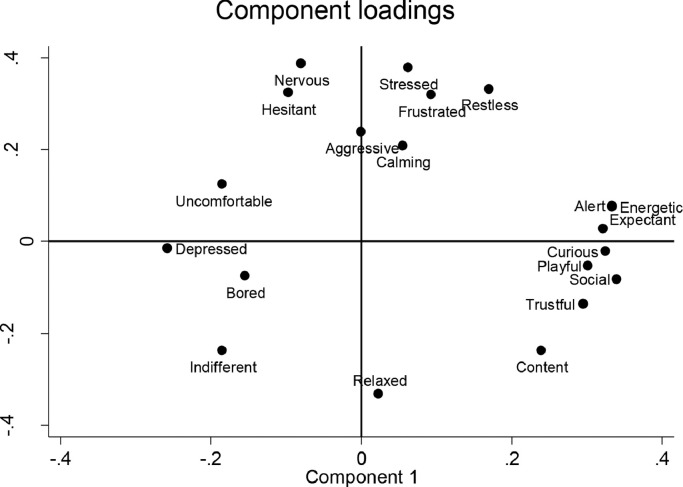
Loading plot depicting how the behavioural terms load along the two main dimensions identified by the principal component analyses of data from the main study (12 videos scored by 22 observers).

The principal component analysis of the data from the 22 observers in the main study also resulted in a two components solution, explaining 34.5% and 21.5% of the variance, respectively. The loading plot in Fig. 2 illustrates the component loadings of each behavioural term across the two components. The first component (PC1) ranged from *depressed, indifferent, uncomfortable* and *bored* to *expectant, energetic, curious* and *sociable*. The second component ranged from *relaxed, content* and *indifferent* to *nervous, stressed, restless* and *frustrated*. Kendall's coefficient of concordance for the first component score was 0.88, indicating high inter-observer agreement. The second component (PC2) had a reliability coefficient of 0.79, also indicating high agreement. Separate analyses for the veterinary students (*n* = 12) revealed that Kendall's *W* for PC1 was 0.89, and PC2 had a reliability coefficient of 0.81. For the veterinary nurse students (*n* = 10), Kendall's *W* for PC1 was 0.88, and PC2 had a reliability coefficient of 0.79 (Table 2).

The twelve veterinary students in the main study achieved high agreement (*W* 0.70–0.89) for six of the behavioural terms (*depressed, restless, alert, sociable, expectant, curious*), while there was moderate agreement (*W* 0.40–0.69) for eleven terms (*content, uncomfortable, playful, relaxed, nervous, hesitant, trustful, energetic, frustrated, indifferent, stressed*), and low agreement for three terms (*bored, aggressive, calming*).

The ten veterinary nurse students in the main study achieved high agreement (*W* 0.70–0.89) for three of the behavioural terms (*depressed, alert, expectant*), moderate agreement (*W* 0.40–0.69) for sixteen terms (*content, uncomfortable, playful, relaxed, restless, bored, sociable, nervous, hesitant, trustful, energetic, frustrated, curious, calming, indifferent, stressed*), and low agreement for one term (*aggressive*).

## 4 Discussion

The aim of this study was to assess the inter-observer agreement of QBA for dogs in a shelter environment. The preferred approach involves using a fixed list of descriptors, as this allows a more standardised and feasible assessment than Free Choice Profiling (Wemelsfelder & Millard, 2009). PCA of the data from both parts of the study supported a two components solution, which overall explained more than 50% of the variance. In the study by Arena and colleagues (2019), four main dimensions were extracted. In our study, PC3 and PC4 also had eigenvalues above 1, but visual inspection of the scree plots suggested that a two components solution was most suitable.

The first component in both parts of this study range from the negative behavioural descriptors *indifferent, depressed, uncomfortable* and *bored*, to positive descriptors, such as *curious, energetic, sociable* and *expectant*, in other words reflecting the dogs’ mood. The second component appears to pertain to arousal, ranging from terms such as *relaxed* or *content* to *nervous, stressed* and *restless*. In the study by Arena et al. (2019), which was published after our data was collected, PC1 characterised *curious*/*playful*/*excitable*/*sociable* demeanour, while PC2 ranged from comfortable/relaxed to *anxious*/*nervous*/*stressed* expression. Although the lists of terms were somewhat different, similar expressive patterns were identified for the first dimensions in both studies.

The Kendall's *W* for PC1 and PC2 were similar in the pilot study compared to the main study, and also between the veterinary students and the veterinary nurse students in the main study. The results suggest that there was a high agreement in how observers assessed the dog's behaviour when using QBA to score video clips, which is in accordance with previous studies applying the QBA approach to shelter dogs (Arena et al., 2017, 2019; Walker et al., 2010). Previous studies in other species have found varying levels of reliability when QBA has been performed using fixed lists (e.g. Bokkers et al., 2012, Czycholl et al., 2017, Diaz-Lundahl et al., 2019, Minero et al., 2016, Muri & Stubsjøen, 2017, Phythian et al., 2013). Arena et al. (2017) suggested that the dog's large expressive repertoire compared to other species, along with thousands of years of domestication and human-dog cohabitation, may have enhanced humans’ ability to interpret dogs’ behaviours and emotions. Studies on pigs and sheep have found satisfactory agreement between observers when scoring videos, but lower agreement when QBA was assessed on-farm (Czycholl et al., 2017; Muri & Stubsjøen, 2017). This may be related to less controlled circumstances concerning exactly what the observers see, potential observer drift (if time has passed since calibration), and/or limited between-farm variation in the animals’ behavioural patterns (Muri & Stubsjøen, 2017). Further research is needed to assess the reliability of QBA for dogs by direct observation.

Varying levels of reliability of individual behavioural terms have been found in previous studies on different species (e.g. Bokkers et al., 2012, Grosso et al., 2016, Minero et al., 2016, Muri & Stubsjøen, 2017). In our study, four terms (*bored, aggressive, calming, hesitant*) showed low reliability (i.e. *W* < 0.40), while the remaining terms showed either moderate or high reliability. Arena et al. (2019) found that 12 out of 20 individual terms showed moderate agreement between 0.50 and 0.60, while 3 terms (*depressed, explorative, aggressive*) fell below 0.50. Aggression has also been difficult for observers to recognise in other studies of dog expressions (Tami & Gallagher, 2009, Bloom et al., 2013). Minero (2016) reached satisfactory agreement for individual descriptors when scoring videos of donkeys, but they reached lower agreement for some terms when assessing on-farm. QBA is considered to be an integrated measure, enabled by the statistical analyses of a number of variables. However, a good reliability on individual terms should be a goal, as this optimises the robustness of the PCA dimensions (Grosso et al., 2016; Muri & Stubsjøen, 2017). The low agreement for the four terms in this study may be due to a low level of these expressions in the video material, or that the assessors found these expressions difficult to assess (Arena et al., 2019), which underlines the importance of thorough training and calibration. Homogeneity in the data is also a known problem in reliability studies. When only a small part of the VAS is used to score a particular behavioural descriptor, even small differences between the observers will have a greater influence on *W*, which was also seen by Muri and Stubsjøen (2017). The dimensions identified by PCA are more robust than each separate behavioural term alone. Given the aim of the method, which is to capture the integrated expressive pattern of the animals’ behaviour, a good agreement on the main dimensions is most important. However, identifying separate terms with low reliability may be of use in the process of developing a list of terms that observers have a common understanding of. In addition, the term's descriptive characterisation or the training material may be improved (Arena et al., 2019). There are no simple criteria for the decision of including or removing terms from a list, but the process must rely on a combination of discussions, agreement testing and training with videos and direct observation (Muri & Stubsjøen, 2017). Some of the terms are partly overlapping, as for example *"depressed" and "uncomfortable".* If all the descriptors correlated poorly, the dimensionality following PCA would be closer to the number of variables, rather than providing us with a few interpretable main dimensions. This is why descriptors that to some degree overlap and correlate are not a problem, but in fact an inherent aspect of the method. Given this, it is difficult to avoid including words in the definition that also are descriptors in their own right.

The current study identified high reliability of QBA when applied by different groups of students, and the IOR was equally high independent of category (senior veterinary students, 3rd year veterinary students and final year veterinary nurse students). The IOR was not only high within the observer groups, but also when data from all observers in the main study were pooled. This indicates that the observers, independent of being veterinary or veterinary nurse students, had a similar way of scoring the videos. The high agreement found between observers in each group suggests that measures are characteristics of the subject animal, rather than characteristics related to the observer (Munch, Wapstra, Thomas, Fisher & Sinn, 2019). However, observers with more varying professional backgrounds and experience may interpret behaviour in different ways, which taps the issue of validity – i.e. to which extent different observers are capable of correctly identifying dog emotional expressions (Arena et al., 2019). Bokkers and colleagues (2012) identified lower levels of reliability in the less experienced group performing QBA of dairy cattle, while Phythian and colleagues Phythian et al., 2013 and Diaz-Lundahl et al. (2019) found high inter-observer agreement for observers with different professional backgrounds when assessing sheep. Duijvesteijn et al. (2014) found low between-observer agreement between farmers, researchers and urban citizens when using QBA to assess video recording of pigs. Munch et al. (2019) found that the agreement between novices and working dog experts was strongly affected by the measurement instrument used to assess dog behaviour. Bloom and Friedman (2013) identified observers’ ability to perceive emotions from a dog's face. They found little difference between experienced and inexperienced people, indicating that learning may not play a large role in the ability to read a dog's emotion via its facial expression. Furthermore, most errors were similar across the experienced and inexperienced groups. They suggested that further research is needed to determine whether humans have innate capacities to recognise emotions in canines, related to the two species’ long shared history or common mammalian ancestry, or if this might be more related to learning. Pongracz, Molnar, Miklosi and Csanyi (2005) suggested that basic emotions and the ability to recognise them is an ancient capability shared by animals and humans. Hence, further research to investigate the IOR of QBA when observers have a greater variation of professional backgrounds and levels of experience is needed.

Duijvesteijn et al. (2014) found QBA to be an effective tool to stimulate mutual learning amongst different stakeholders when assessing videos of pigs. In order to stimulate discussions, questions were asked about what influenced the scorings, what was understood by the different QBA terms, and whether and why they considered certain terms relevant for pig welfare. Similarly, QBA can be used as a learning tool for students. Old and Spencer (2011) proposed that learning activities developed to increase the awareness of animal behaviour should be provided prior to hands-on live animal practical sessions in animal-associated educations. QBA may be a useful tool to emphasise the importance of careful observation of the animals’ body language, in order to improve both student handling skills, safety and animal welfare.

The practical relevance of QBA may be extended to animal care situations, where identification of shifts in animal expression, as and when they happen, is the main concern (Wemelsfelder, Young & Martyniuk, 2016). Good health is an essential part of animal welfare. Lameness has been found to be correlated with QBA scores in sheep, suggesting that compromised health had a deleterious effect on the sheep's emotional state (Phythian et al., 2016). Dairy cattle in the acute phase of E. coli mastitis were also interpreted to be in a negative emotional state, as assessed with QBA (des Roches et al., 2018). Hence, in conjunction with other physiological and behavioural indicators, QBA could potentially be used as a tool to identify dogs with disease or pain issues, which compromise the welfare to a degree where the animals’ emotional state is visibly affected.

## 5 Conclusions

We conclude that QBA and the terms included in the fixed list in this study were reliable for assessing video recordings of shelter dogs when applied by both veterinary students and veterinary nurse students. This method may also be useful as a learning tool for students. Our results support further exploration of the reliability and validity of applying this method to the assessment of shelter dog welfare when scoring animals live in a practical setting.

## Ethical statement

This research does not involve experimentation on animals. The dogs in this study were video-recorded in their kennels with minimum disturbance of the animals. The study was conducted in accordance with the Ethical Guidelines for the Use of Animals in Research (The Norwegian National Committee for Research Ethics in Science and Technology (NENT), 2018). A written informed consent was obtained from the administration of the shelter prior to the study. All students who assessed the videos gave their informed consent for participation prior to the study.

## Funding

The project was funded by Smådyrpraktiserende veterinærers forenings vitenskapelige og faglige fond (Norwegian Small Animal Veterinary Association's Scientific Foundation) and the Norwegian Veterinary Institute's internal funding (Strategic programme on animal welfare).

## Declaration of Competing Interest

The authors declare no conflicts of interest.
